# Diurnal Variation, Topographical Distribution and Day-to-Day Repeatability of Ocular Surface Epithelial Immune Cells in Individuals with Dry Eye Disease †

**DOI:** 10.3390/jcm15072582

**Published:** 2026-03-27

**Authors:** Soumen Sadhu, Isabelle Jalbert, Blanka Golebiowski, Fiona Stapleton

**Affiliations:** Optometry Clinic, School of Optometry and Vision Science, University of New South WalesSydney, Level 3, North Wing, RMB, Gate 14, Barker Street, Sydney, NSW 2052, Australia; s.sadhu@unsw.edu.au (S.S.); i.jalbert@unsw.edu.au (I.J.); b.golebiowski@unsw.edu.au (B.G.)

**Keywords:** dry eye disease, in vivo confocal microscopy, ocular surface epithelial immune cell, diurnal variation, topographical differences

## Abstract

**Objectives**: To assess diurnal changes, topographical differences, and day-to-day repeatability of ocular surface epithelial immune cell (EIC) density and morphology in dry eye disease (DED). **Methods**: Sixteen participants with moderate-to-severe DED (mean (SE) age 49.4 (4.2) years) underwent in vivo confocal microscopy at three timepoints (day-1 morning and evening and day-2 morning) at six locations: central cornea, inferior whorl, inferior cornea, and temporal cornea, limbus and conjunctiva. Diurnal and topographical variation in EIC density and morphology were analyzed using linear mixed-effects models with adjusted pairwise comparisons. Day-to-day repeatability was assessed using the coefficient of repeatability (CoR) for density and Cohen’s kappa for morphology. **Results**: EIC density and morphology varied by location (*p* < 0.001) but not by timepoint at any location (*p* = 0.59–0.90). Density was highest at the inferior cornea (model-estimated mean: 101.2 (SE: 21.7) cells/mm^2^) and temporal limbus (104.3 (22.7) cells/mm^2^), and lowest at the central cornea (26.8 [5.1] cells/mm^2^ and inferior whorl (38.3 [8.2] cells/mm^2^; all pairwise, *p* < 0.001). EICs with large bodies were more frequent in conjunctiva (100%), inferior cornea (94%), and temporal cornea (87%), than in central cornea (34%) and whorl (19%) (all *p* ≤ 0.007). EICs with dendrites, and with long dendrites were similarly distributed (*p* < 0.001). Bland–Altman analysis showed low mean bias and EIC density was more repeatable at the central (CoR ± 23.8 cells/mm^2^) and temporal cornea (±27.5 cells/mm^2^) than the inferior cornea (±47.9 cells/mm^2^) or temporal conjunctiva (±42.3 cells/mm^2^). Morphology agreement was substantial to near-perfect (κ = 0.71–0.97). **Conclusions**: In DED, EIC density and morphology are diurnally stable and maintain topographic distribution patterns similar to healthy eyes. Day-to-day repeatability show location dependent patterns. The study provides feasibility data for using IVCM for EIC metrics in disease monitoring.

## 1. Introduction

The immune system plays a significant role in the pathogenesis of dry eye disease (DED) [1,2,3,4]. Cells of both the innate and the adaptive immune systems such as the dendritic cells, macrophages and T lymphocytes are upregulated at the ocular surface in DED [1,4,5]. Increased levels of several proinflammatory cytokines and chemokines released by these immune cells are observed in the tears, and this likely drives ocular surface inflammation [2,4,6,7,8]. Diurnal and nocturnal variations in tear film composition, tear cytokine profiles and in DED clinical signs and symptoms have previously been established [9,10,11,12]. Diurnal variations in DED symptoms are frequently reported, with a subset of the DED population experiencing more frequent symptoms either in the morning or evening [13]. Symptom severity can range from mild to severe, with corresponding diurnal variations in visual function [13,14]. Signs of DED such as conjunctival redness, corneal staining, tear osmolarity, and tear evaporation rate similarly fluctuate over the course of the day [15,16,17,18,19,20].

Inflammatory tear cytokines such as IL-6, IL-1ra, IL-10, and IL-1β exhibit significant fluctuations in healthy individuals [10,11,16]. These cytokines are often secreted by ocular surface epithelial cells and immune cells in response to desiccating stress and have been linked to DED pathogenesis [21]. Some of these molecules show higher tear concentrations in the evening and in the morning after sleep, indicating that their tear levels follow a diurnal pattern [9,10,11,16]. These fluctuations pose significant challenges in the diagnosis and grading of severity in DED.

There is thus a growing interest in developing robust DED biomarkers. The number of activated central corneal dendritic cells could serve as a biomarker for diagnosing DED, as well as distinguishing between autoimmune and non-autoimmune forms of DED [22]. Understanding the diurnal behaviour of ocular surface immune cells is likely to be important, particularly when considering their potential utility as biomarkers of DED. In vivo confocal microscopy (IVCM) is a valuable tool for evaluating corneal epithelial immune cells (EIC) in various ocular surface diseases and for monitoring the effects of DED treatment [23,24,25,26,27].

Previous studies of healthy individuals reported no significant diurnal variation in epithelial dendritic cells in the central cornea [28,29], peripheral cornea and limbus [29]. Topographical differences in EIC density and morphology are reported in healthy individuals [27,29,30,31,32], with higher EC density in the inferior, nasal, and temporal peripheral corneal and limbal locations compared to the central cornea and inferior whorl [29,30,32]. This topographical distribution pattern was also observed in participants with allergic conjunctivitis, where the corneal limbus and the peripheral cornea showed higher EIC density and morphological features compared to the central cornea and the inferior whorl [30].

Systemic inflammatory conditions can alter normal diurnal immune rhythms. For example, an exaggerated nocturnal rise in IL-6 was observed in rheumatoid arthritis, but is not observed in healthy individuals and is closely linked to morning stiffness [33,34]. Accentuated nighttime airway inflammation occurs in asthma patients compared to healthy individuals [35]. Chronic inflammation can amplify or disrupt normal circadian immune rhythms, suggesting the possibility that diurnal patterns of ocular surface immune cells could be altered in DED.

Although central corneal EIC density is increased in DED compared to healthy controls, there is a wide variation among individuals with DED, with reported density values ranging from 11.2 to 250 cells/mm^2^ [36]. A similarly broad range in EIC density is also observed in healthy controls [37]. This heterogeneity may be influenced by DED severity, autoimmune or systemic involvement, and methodological differences between studies. Given that these immune cells are motile and exhibit dynamic changes over time, the variations may also reflect underlying temporal fluctuations in EIC behaviour [38,39]. A better understanding of the temporal variation and topographical distribution of ocular surface EICs in the disease state could inform the utility of EIC characteristics as a biomarker for DED diagnosis and treatment monitoring. Recognizing and accounting for normal diurnal fluctuations may also enhance the interpretation of clinical trial results and the assessment of anti-inflammatory therapies.

Given that DED signs, symptoms, tear composition, and inflammatory markers show diurnal variation, and that healthy eyes exhibit topographical differences in EIC patterns at the ocular surface, it remains unclear whether these patterns are preserved or altered in DED and how stable they are diurnally. This study was designed as a confirmatory/feasibility study to confirm whether location-specific EIC patterns observed in healthy eyes are preserved or altered in DED and to establish short-term stability and repeatability parameters of EIC density and morphology to inform future studies and clinical trials.

The primary aims of this study were to assess the following in DED:

1. Diurnal variation in EIC density and morphology at different ocular surface locations.
2. Topographical distribution of EICs across the ocular surface.
3. Day-to-day repeatability of EIC density and morphology at each location.

The secondary aim was to assess the associations between EIC density and DED signs and symptoms.

## 2. Materials and Methods

This prospective cross-sectional study was approved by the Human Research Ethics Committee of the UNSW Sydney, Australia (iRECS5817), and followed the principles of the Declaration of Helsinki. After obtaining written informed consent, participants were screened for eligibility using the Ocular Surface Disease Index (OSDI) questionnaire and relevant signs to establish a diagnosis of DED. The inclusion criteria were age of 18 years and above, and OSDI ≥13 and with any one of the following signs: (i) non-invasive tear break-up time (NIBUT) <10 s, (ii) Oxford staining grade ≥1, and (iii) tear meniscus height (TMH) <0.20 mm [40]. Participants who were using systemic anti-inflammatory therapy for an underlying autoimmune disease must not have changed their medications within the past 3 months. Participants were excluded from the study if they had used anti-inflammatory eye drops within the past 4 weeks or if DED was secondary to Stevens-Johnson syndrome, cicatricial conjunctival disease, or associated with the use of topical medications for glaucoma. Additional exclusion criteria included a history of ocular surface procedures or ocular surgery within the past 6 months; contact lens use within the past 4 weeks; pregnancy, breastfeeding, or childbirth within the past 3 months; and the presence of active intraocular inflammation.

The sample size for this study was calculated for repeated-measures ANOVA (within-subject factors) using G*Power 3.1. An effect size of *f* = 0.5 was calculated based on an expected within-subject difference in EIC density of 12 cells/mm^2^ between visits, with a standard deviation of 15 cells/mm^2^ and an assumed correlation coefficient of *r* = 0.6 (ε = 1.0) between repeated measures [29]. The sample size calculation indicated that 15 participants would be sufficient to achieve 80% power at a 5% significance level.

### 2.1. Clinical Procedures

The intensity of DED-related symptoms—dryness, eye pain, burning sensation, foreign body sensation, vision quality, fluctuating vision, itching and overall discomfort—was rated using a numerical rating scale (NRS, ranging from 0 to 100) [41]. One eye was randomly selected as the study eye. There were three visits: day 1 morning visit (between 8:00 and 9:30), evening visit (between 18:00 and 20:00) and day 2 morning visit (between 8:00 and 9:30). All participants were examined within 2 h of waking in the morning.

The following DED tests were sequentially performed during all visits: non-invasive TMH and NIBUT were measured using the Keratograph 5M (K5M; Oculus Optikgeräte, GmbH, Wetzlar, Germany). Fluorescein tear film break-up time (FBUT) was measured following the instillation of sodium fluorescein (Bausch & Lomb, Sydney, NSW, Australia), moistened with saline. Ocular surface staining was then assessed using sodium fluorescein and lissamine green (Bausch & Lomb, Sydney, NSW, Australia), applied to the lower palpebral conjunctiva. Staining of the ocular surface was graded using the Oxford grading schema, from 0 to 5 [42]. TMH was measured at the 6 o’clock position. For NIBUT & FBUT, the average of 3 readings for the first breakup was used for analysis.

Signs of meibomian gland dysfunction were assessed using the Meibomian Glands Yielding Liquid Secretion (MGYLS) score [43] and Meibomian Gland Secretion (MGS) score in the first visit only. These were measured by applying digital pressure to the lower tarsal plate for 10–15 s to observe the number of expressible glands among the central 8 glands of the lower eyelid (MGYLS score range: 0–8). The MGS was graded from 0 to 3, where grade 0 indicated clear meibum fluid, grade 1 indicated cloudy liquid, grade 2 indicated cloudy particulate fluid, and grade 3 indicated inspissated toothpaste-like fluid. The highest secretion grade observed among the 8 central glands was recorded (MGS score range: 0–3) [43,44].

**Figure 1:**
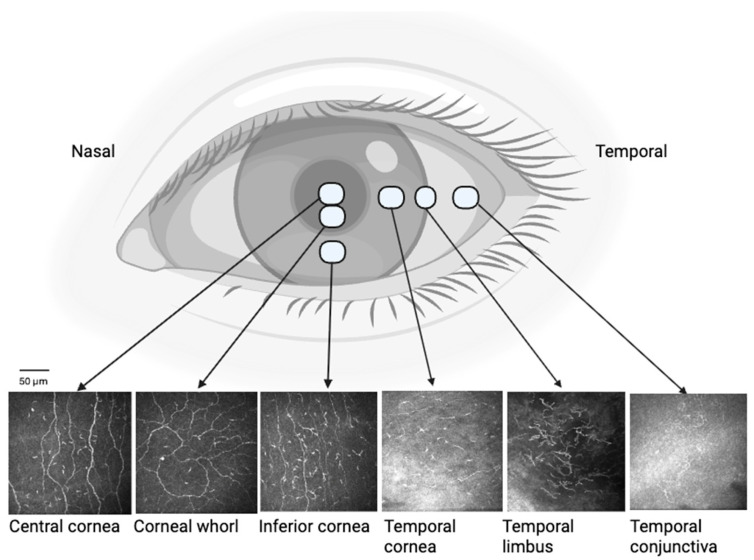
Diagram illustrating the six ocular surface topographical regions assessed using in vivo confocal microscopy. Representative 400 × 400 μm images are shown for each ocular surface site. Scale bars represent 50 μm.

IVCM (Heidelberg Retinal Tomograph III Rostock Corneal Module, Heidelberg, Germany) using the 63× objective lens was performed at six ocular surface locations: central cornea, inferior whorl, inferior cornea (approximately 2–3 mm inside the inferior limbus at 6 o clock position), temporal cornea (approximately 2–3 mm inside the temporal limbus at 9 o clock position), temporal limbus (9 o clock position) and temporal bulbar conjunctiva (3 mm outside the temporal limbus) (Figure 1). Field of view was 400 × 400 µm (384 × 384 pixels; lateral resolution ≈1–2 µm; axial resolution ≈4 µm). Section scanning mode was used with a standardized gain/contrast setting that was kept constant across all visits and locations. Imaging was conducted during all three visits following the application of unpreserved topical anaesthetic eye drops (oxybuprocaine hydrochloride 0.4%, Minims, Bausch & Lomb, Kingston, UK). A sterile disposable tomocap (Heidelberg, Germany) was attached in front of the IVCM objective lens and a carbomer gel (Viscotears, Alcon, Sydney, NSW, Australia) was used as a coupling agent. The tomocap was aligned at different ocular surface locations, while the participant fixated a red-light target in the contralateral eye. This fixation light was adjusted centrally, superiorly, inferiorly and temporally to scan the central cornea, inferior cornea, inferior whorl and temporal cornea, limbus and conjunctiva respectively. The microscope was focused manually on the corneal sub-basal nerve plexus at a depth of 25–65 µm from the superficial corneal epithelium, where immune cells are optimally visualized [36]. The corneal locations were identified based on the pattern and orientation of the corneal nerves. The inferior corneal whorl was identified first, and images were captured, followed by the remaining corneal locations. The central cornea was imaged approximately 2–3 mm above the corneal whorl. The limbal cornea was identified by the typical extensions of the palisades of Vogt and highly pigmented areas distinct from the corneal epithelium. The inferior cornea was imaged where the nerves are vertically oriented and 2–3 mm inside the inferior limbus. Similarly, the temporal cornea was imaged where nerves run horizontally.

In the limbal cornea, EICs were identified at a depth of 45–80 µm and at 5–25 µm in the conjunctival epithelium. Approximately 50 images were captured at each location using the “section mode” in the Heidelberg software (Heidelberg Eye Explorer, version 1.9; Heidelberg Engineering, Heidelberg, Germany). To enhance consistency in imaging across visits, reference images from the first visit were used to guide image acquisition for corneal locations and at fixed depth ranges from the superficial epithelium. The identification of the pattern and orientation of sub-basal nerves was employed to approximate the same imaging location for subsequent visits. As an operator aid, approximately two consecutive 180° turns of the HRT position drum were used when moving from the inferior whorl toward the central, inferior and temporal corneal locations to approach the target region. However, final positioning was confirmed by sub-basal nerve pattern/orientation from the previous reference images to scan the same location. Visual cues from the confocal camera image were also used during repositioning. Immune cells observed via IVCM are often labelled as “Langerhans cells” or “dendritic cells.” However, in this study, we refer to them more broadly as epithelial immune cells (EICs). This terminology reflects the inherent limitation of IVCM, which does not allow for direct phenotypic identification of cells. The precise identity of corneal immune cells remains a point of debate, though recent advances in the field suggest that the healthy cornea contains both intraepithelial lymphocytes and dendritic cells [36,38,45,46]. When the ocular surface is disturbed (as, for example, in DED), dendritic cells are likely activated and may present antigens to T cells, contributing to T cell recruitment and proliferation within the cornea [4].

### 2.2. Image Analysis for EIC Density and Morphology

Images were screened against pre-specified quality criteria: (i) clear focus (clearly defined sub-basal nerve patterns and bright EIC), (ii) adequate contrast, (iii) absence of motion blur, compression artifacts, and pressure lines, and (iv) ≤20% overlap between selected frames (assessed by nerve pattern matching). A single investigator (SS), masked to the study visit and participant DED status, manually selected and analyzed the IVCM images. Selection and counting were performed in a randomized, masked manner. Five, best focused and less than 20% overlapped images were selected for each location to quantify EIC density and to grade EIC morphology. For the inferior whorl, only one representative image was analyzed. During image selection, to enhance between-visit consistency at corneal sites, Day 1 frames were used as visual references for Day 1 evening and Day 2 morning visits, and frames were chosen by matching the sub-basal nerve pattern/orientation observed in the Day 1 reference images. If an exact pattern match was not found, the nearest frames along the same horizontal plane were selected to minimise positional/depth variability in EIC parameters. For limbal and conjunctival sites, the five best quality, non-overlapping images were selected at each timepoint.

Manual counts were performed in ImageJ (version 1.54p; https://imagej.net/ij/; 25 August 2025) software calibrated for 400 × 400 µm frames (area 0.16 mm^2^). Calibration was done for 384 × 384 pixels = 400 µm, yielding a pixel size of ~1.04 µm; the scale was set and fixed prior to counting for all locations and participants.

**Figure 2:**
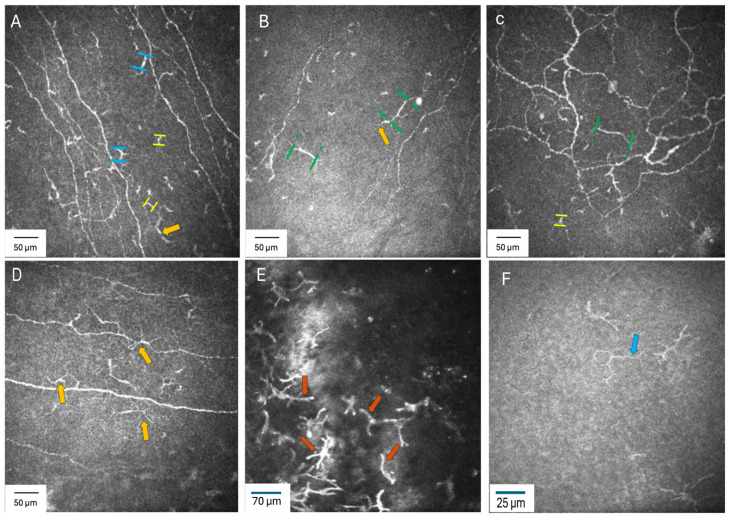
Categorization of epithelial immune cell (EIC) morphology. Representative in vivo confocal microsopy images—(**A**) (400 × 400 µm) central cornea, (**B**) inferior cornea, (**C**) corneal whorl, (**D**) temporal cornea, (**E**) temporal limbus and (**F**) temporal conjunctiva—show examples of small EIC body size (10–25 µm, yellow dash, **A**,**C**), medium EIC body size (26–40 µm, blue dash, **A**) and large EIC body size (>40 µm, green dash, **B**,**C**), presence of dendrites (orange, blue and red arrows, **B**–**F**), presence of long dendrites (orange arrows in **A**–**C** and blue arrow in **F**) and presence of thick dendrites (red arrows, **E**) [49].

Ocular surface EICs were defined as “bright corpuscular or specular, highly reflective bodies with and without dendrites with a diameter of 10–65 µm located at the level of sub-basal nerve plexus about 5–65 µm deep” [23,36,47,48]. The number of cells within each 400 × 400 µm frame was manually counted using the ImageJ “Analyze” and “Measure” functions after calibration. The diameter of each hyper-reflective presumed immune cell was measured, and cells falling within the 10–65 µm range were counted as EICs. Cell density was expressed as cells/mm^2^ (Figure 2). The mean value of five images was used for EIC density. EIC morphology was determined using a published grading system for EIC body size, presence of dendrites, presence of long dendrites and presence of thick dendrites [49]. EIC body size was graded as small (10–25 µm), medium (26–40 µm) and large (>40 µm); and the largest cell body observed in any of the five selected images was recorded. Similarly, the presence of dendrites, long dendrites, and thick dendrites was graded as a binary variable (yes/no), with a “yes” assigned if the feature was present in at least one of the five selected images [49]. Representative IVCM images of the EIC morphological grading system are illustrated in Figure 2. The manual counting approach for EIC density and the morphology grading used in this study follow established procedures previously validated [30,49]. Intra-observer and inter-observer repeatability of these methods, using identical methods, have been reported with good reproducibility, supporting the reliability of the manual assessment approach employed in the current work [30,49].

### 2.3. Data Analysis

Data were analyzed using R 4.4.1 software (https://posit.co/products/open-source/rstudio/; accessed on 10 September 2025). Normality for EIC density data was assessed, and log transformation was applied where data were not normally distributed after adding 0.5 to EIC density values of “zero”. Model assumptions were verified by confirming normal distribution of residuals (Shapiro-Wilk test: W = 0.99, *p* = 0.43) and Q-Q plots showing appropriate fit. EIC body size (ordinal—small, medium and large) and EIC dendritic characteristics (binary—yes/no) were expressed as percentages. A linear mixed-effects model was used to assess diurnal variation in EIC density and DED parameters at all locations (data from day 1 morning and evening timepoints were included in the model). All mixed-effects models included location and timepoint as fixed effects, and participants as a random effect to account for repeated measures and for multiple comparisons. All mixed-effects models utilized the lme4 and lmerTest or clmm packages in RStudio (R 4.4.1). Statistical significance was set at *p* < 0.05. Actual means, model-estimated means for mixed-model analysis and standard error (SE) were reported for EIC density and DED data, unless specified. For pairwise comparisons between locations, aggregated model-estimated means from the two timepoints (day 1 morning and evening) were used. Mean differences in EIC density between locations were adjusted for multiple comparisons and the adjusted *p*-values were reported.

### 2.4. Effect of Time and Location on EIC Density

To examine the overall effect of time, location and their interaction (timepoint × location) on EIC density, a linear mixed-effects model was utilized. Post hoc pairwise comparisons of the model-estimated marginal means (emmeans) between timepoints and between six ocular surface locations were conducted using *t*-tests with Tukey’s adjustment. Model-estimated EIC density means and SEs were calculated by back-transforming from the log scale.

### 2.5. Effect of Time and Location on EIC Morphological Parameters

To assess the overall effects of time, location and their interaction on EIC body size, an ordinal logistic regression through cumulative link mixed models was used with Type II Wald chi-square tests (χ^2^). Post hoc pairwise comparisons between locations were conducted using emmeans using Wald z-tests, with Tukey’s adjustments. For EIC dendrite characteristics (presence of dendrites, long dendrites, and thick dendrites), a generalized linear mixed models with binomial distribution analyses were conducted to assess the overall effect of time, location, and their interactions with Type II Wald chi-square tests (χ^2^). Post hoc pairwise comparisons of emmeans were conducted using Wald z-tests with Tukey’s adjustment.

### 2.6. Day-to-Day Repeatability

Day-to-day repeatability of EIC density at six locations was assessed using Bland–Altman plots and coefficient of repeatability (CoR) [50]. Participants with an EIC density of “zero” at either Day 1 or Day 2 for a given location were excluded from the repeatability analysis. The mean differences between day 1 morning vs. day 2 morning measurements were calculated, and Wilcoxon signed-rank tests were performed to detect significant bias. The CoR was calculated as 1.96 × standard deviation of the differences, with relative repeatability coefficient (CoR%) expressed as a percentage of the mean measurement value for each location. Day-to-day agreement for EIC body size was calculated using Cohen’s weighted kappa and unweighted Cohen’s kappa for the presence of dendrites, presence of long dendrites, and presence of thick dendrites. Morphology repeatability analysis was conducted, where the morphology findings were available at both visits. McNemar’s test was applied to binary outcomes to assess systematic disagreement.

### 2.7. Association Between EIC Density and DED Parameters

Associations between EIC density (dependent variable) and DED parameters including NIBUT, FBUT, ocular staining grade, TMH, MGS score, MGYLS score, OSDI score, and individual NRS symptom domains were evaluated using separate linear mixed-effects models for each parameter. Each model included the DED parameter, location, and their interaction as fixed effects. Where the parameter × location interaction was significant, location-specific associations were reported, and where no interaction was observed, only the overall associations were presented. All three timepoints were pooled for all parameters, except for the OSDI score, which was assessed only at the first visit. Participant was included as a random effect to account for repeated measures across locations and timepoints. The significance of fixed effects was evaluated using *t*-tests with Bonferroni-adjusted *p*-values to account for multiple comparisons. Standardized regression coefficients (β) and adjusted *p*-values were reported.

## 3. Results

### 3.1. Participant Characteristics

Sixteen participants with DED (56% females, median age of 45.5 years (IQR: 38.0–66.2 years) were enrolled in the study. The median duration of DED was 48.0 (36.0–61.0) months. Median OSDI score was 50.8 (37.8–52.7), NIBUT 3.5 (2.5–5.0) seconds, FBUT 3.0 (2.0–4.0) seconds, central TMH 0.17 mm (0.14–0.203 mm), and Oxford staining grade 1.5 (0.4–1.9). A detailed description of participant demographics and DED parameters is provided in Supplementary Table S1. All participants reported using one or more lubricating eye drops. There was no significant effect of time of day for NIBUT (F = 0.08; *p* = 0.21), FBUT (F = 0.04; *p* = 0.81), central TMH (F = 0.07; *p* = 0.47), and Oxford staining grade (F = 0.04; *p* = 0.80). Although 8 of 16 participants reported more severe DED symptoms in the evening, the NRS scores for symptom domains, including dryness, vision quality, fluctuating vision, itching, burning sensations, foreign body sensation, eye pain, and overall discomfort, did not show a significant effect of time of day (all *p* ≥ 0.15).

EICs were observed in the central cornea, inferior whorl, and inferior cornea of all participants at all timepoints. EICs were observed at all timepoints in fifteen participants in the temporal cornea and temporal limbus, and in twelve participants in the temporal conjunctiva. At day 1 morning the actual median (IQR), central corneal EIC density was 27.5 (15.6–53.3) cells/mm^2^; inferior whorl 44.5 (30.5–76.6) cells/mm^2^; inferior cornea 100.5 (51.8–131.9) cells/mm^2^; temporal cornea 40.0 (20.8–60.7) cells/mm^2^; temporal limbus: 101.0 (67.5–123.7) cells/mm^2^; and temporal conjunctiva: 38.8 (11.0–64.4) cells/mm^2^ (Supplementary Table S2).

### 3.2. Diurnal Variation in EIC Density and Morphology

#### 3.2.1. EIC Density

**Figure 3:**
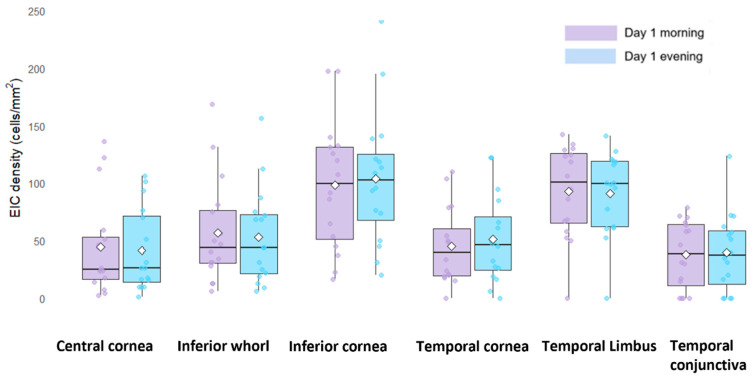
Diurnal variation and topographical differences in epithelial immune cell density (cells/mm^2^) in 16 participants with dry eye disease. Footnotes: Plots represent data points for actual data (not log-transformed data). Dots represent individual data, with the central black line indicating the median and boxes showing the interquartile range (25th–75th percentile), lower and upper extremes and outliers lying above and below Q3 ± 1.5 × interquartile range. White diamond symbols show the mean values of the actual data.

**Figure 4:**
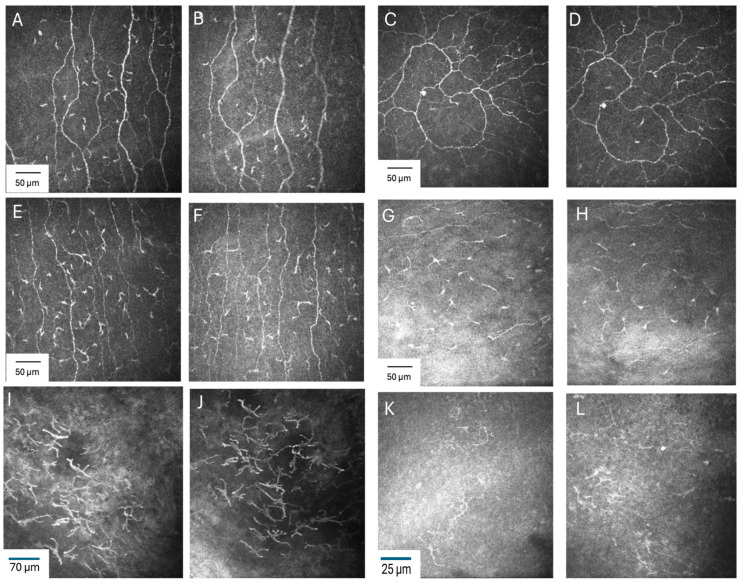
Representative in vivo confocal microscopy images of epithelial immune cells (EIC) obtained from a dry eye participant at two timepoints (Day 1 morning and evening) across six ocular surface locations. Central cornea (**A**,**B**), inferior (**C**,**D**), inferior cornea (**E**,**F**), temporal peripheral cornea (**G**,**H**), temporal limbus (**I**,**J**), and temporal bulbar conjunctiva (**K**,**L**). Across all locations, there were no significant changes observed in EIC density and morphology between morning and evening visits.

The linear mixed-effects model did not show an effect of time of day on EIC density (F = 0.01; *p* = 0.90) and this was consistent across all locations and at both timepoints (timepoint x location interaction; F = 0.10; *p* = 0.99). Comparison between day 1 morning and evening measurements showed no significant difference (mean difference [MD]: 0.3; standard error [SE]: 0.2) in the central cornea (Table 1). Post hoc pairwise comparisons at all six ocular surface locations also showed that there were no significant differences between the two timepoints (all *p* ≥ 0.67) (Table 1). The model-estimated EIC density means and SEs for morning and evening timepoints at each location are provided in Table 1 and the distribution of the actual EIC data (mean, media and interquartile range (IQR)) by timepoint and location is illustrated in Figure 3. Figure 4 shows the representative IVCM images of six locations on the day 1 morning and evening visit of a dry eye participant.

#### 3.2.2. EIC Morphology

There was no significant diurnal variation in EIC body size (χ^2^ = 0.02, *p* = 0.88) and this was consistent across all ocular surface locations (interaction: χ^2^ = 5.1, *p* = 0.40). Similarly, there was no significant diurnal variation in presence of dendrites (χ^2^ = 0.28, *p* = 0.59), presence of long dendrites (χ^2^ = 0.00, *p* = 0.99) or presence of thick dendrites (χ^2^ = 0.38, *p* = 0.94) and this was consistent across all ocular surface locations (all *p* ≥ 0.90) (Supplementary Table S3).

### 3.3. Topographical Differences in EIC Density and Morphology

#### 3.3.1. EIC Density

There was a significant effect of location on EIC density (F = 19.30, *p* < 0.001). Comparisons between locations showed the highest EIC density at the temporal limbus (model-estimated mean: 104.4 cells/mm^2^, SE: 22.6) and inferior cornea (mean: 101.4 cells/mm^2^, SE: 21.5) compared to other locations (all *p* ≤ 0.001; Table 1). EIC density at the temporal limbus was higher than the central cornea (MD: 77.8 cells/mm^2^, SE: 23.3; *p* < 0.001), inferior whorl (MD: 66.0 cells/mm^2^, SE: 24.3; *p* < 0.001), temporal conjunctiva (MD: 61.2 cells/mm^2^, SE: 24.9; *p* < 0.001), and temporal cornea (MD: 62.5 cells/mm^2^, SE: 24.8; *p* < 0.001). EIC density was higher in the inferior cornea than the inferior whorl (MD: 62.9 cells/mm^2^, SE: 23.2; *p* < 0.001) and temporal cornea (MD: 59.4 cells/mm^2^, SE: 23.7; *p* < 0.001) (Supplementary Table S4). At both timepoints the lowest density was found at the central cornea (mean: 26.3 cells/mm^2^, SE: 5.0) compared to the inferior cornea (MD: −74.7 cells/mm^2^, SE: 22.3; *p* < 0.001) and the temporal limbus (MD: −77.8 cells/mm^2^, SE: 23.3; *p* < 0.001).

#### 3.3.2. EIC Morphology

**Figure 5:**
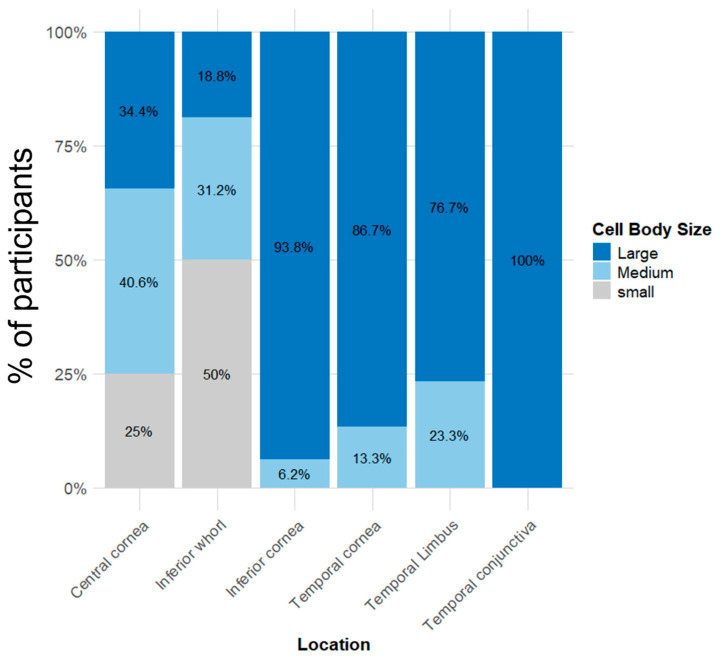
Epithelial immune cell body size by ocular surface location in 16 participants with dry eye disease. Footnote: For each location, the percentages of large, medium, and small cells represent the average of the percentages observed during the day 1 morning and evening visits.

**Figure 6:**
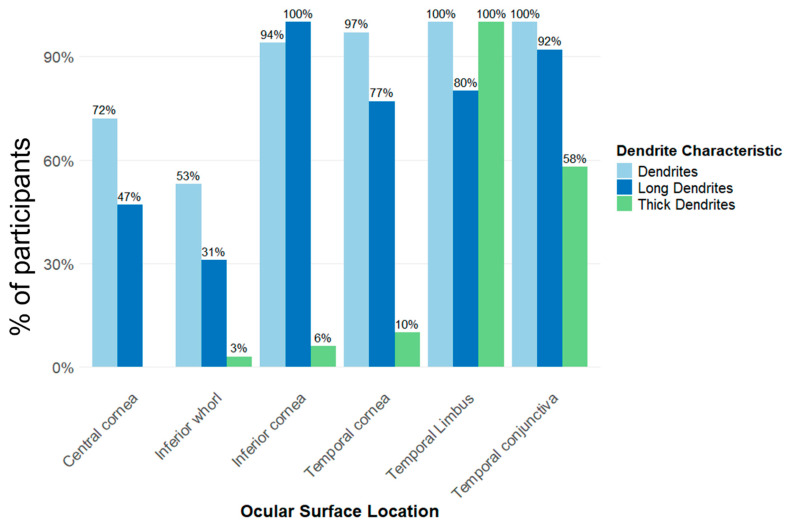
Epithelial immune cell dendrite characteristics by ocular surface locations in 16 participants with dry eye disease. Footnote: For each location, the percentages of cells represent the average of the percentage observed during the day 1 morning and evening visits.

There were significant differences in the topographical distribution of EIC body size across the ocular surface (χ^2^ = 94.3, *p* < 0.001). A higher proportion of large EICs were observed in the temporal conjunctiva (100% large cell body size), inferior cornea (94%), temporal cornea (87%) and temporal limbus (77%) compared with the central cornea (34%) and inferior whorl (19%) (all pairwise comparisons *p* < 0.001; Supplementary Table S5). Figure 5 shows the percentage distribution of EIC body size across ocular surface locations. There was a significant effect of location for the presence of dendrites (χ^2^ = 16.0, *p* = 0.004). There was a higher proportion of presence of dendrites at the temporal conjunctiva (100%), temporal limbus (100%), temporal cornea (97%), and the inferior cornea (94%) compared to the inferior whorl (53%; all *p* ≤ 0.03). Similarly, there was a significant location effect for the presence of long dendrites (χ^2^ = 26.5, *p* < 0.001). There were higher proportion of presence of long dendrites at the inferior cornea (100%), temporal conjunctiva (91.7%), temporal limbus (84.5%), and the temporal cornea (76%) than central cornea (45%; all *p* ≤ 0.04) and inferior whorl (30.0%; all *p* ≤ 0.003) (Figure 6 and Supplementary Table S6). There was a significant location effect for the presence of thick dendrites (χ^2^ = 18.2, *p* = 0.002), with higher proportions at the temporal limbus (100%) and temporal conjunctiva (58%). Figure 6 shows the percentage distribution of epithelial immune cell dendritic characteristics across ocular surface locations.

### 3.4. Day-to-Day Repeatability

#### 3.4.1. EIC Density

**Figure 7:**
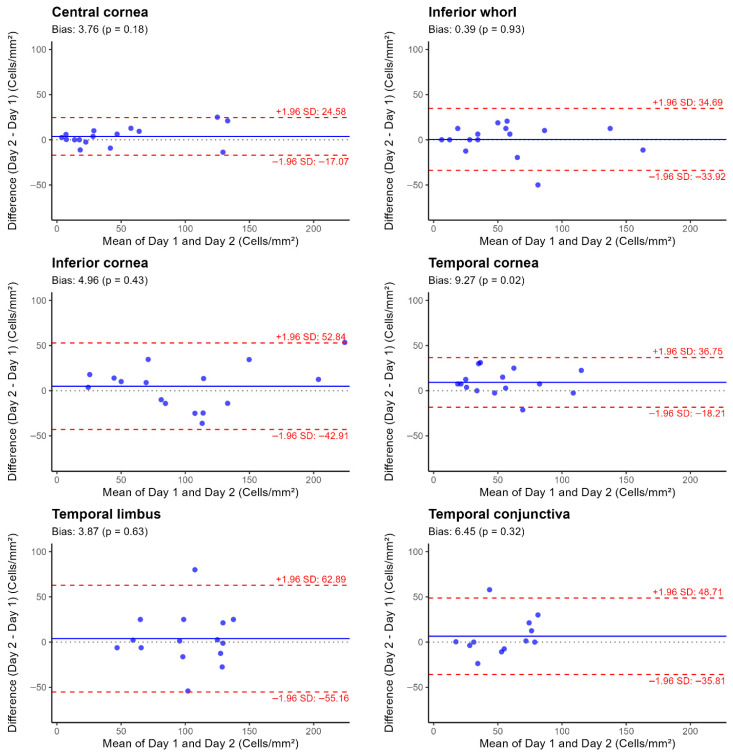
Bland–Altman plots illustrating the day-to-day repeatability in epithelial immune cell density measurements across six ocular surface locations in 16 participants with dry eye disease. Footnote: Each plot shows the difference between Day 1 and Day 2 measurements (y-axis) against their mean (x-axis), with the mean bias (solid line) and 95% limits of agreement (LOA) (±1.96 × SD LoA) (dashed lines).

Day-to-day repeatability of EIC density varied across ocular surface locations. In terms of CoR values, measurements were more repeatable in the central cornea (CoR = ±23.8 cells/mm^2^; CoR% = 44.6%), temporal cornea (±27.5 cells/mm^2^; 47.3%), and inferior whorl (±34.3 cells/mm^2^; 51.0%) than the temporal conjunctiva (±42.3 cells/mm^2^; 77.8%), inferior cornea (±47.9 cells/mm^2^; 43.8%), and temporal limbus (±59.0 cells/mm^2^; 61.2%). Mean bias and 95% limits of agreement (LoA) between day 1 and day 2 measurements for each location are shown in Figure 7.

#### 3.4.2. EIC Morphology

The overall (all locations combined) day-to-day agreement for EIC body size was substantial [51], with a weighted Cohen’s kappa of 0.72. Day-to-day agreement for EIC body size was lowest in the central and inferior cornea (both 69% agreement between day 1 and day 2 morning), while all other locations showed agreement of 88% or higher. Day-to-day agreement ranged from substantial for the presence of dendrites (Cohen’s kappa = 0.71), long dendrites (Cohen’s kappa = 0.76), to near perfect for thick dendrites (Cohen’s kappa = 0.97), and thin dendrites (Cohen’s kappa = 0.94). All locations showed ≥80% observed agreement between days for all three dendritic characteristics. No significant bias between day 1 and day 2 morning agreements was observed for any parameter (all McNemar *p* ≥ 0.65).

### 3.5. Associations Between EIC Density and DED Parameters

There were positive associations between ocular surface staining grade and EIC density in the central cornea (Bonferroni-adjusted for six locations, β = 0.64, *p* = 0.004), inferior whorl (β = 0.82, *p* < 0.006), inferior cornea (β = 0.45, *p* < 0.006), and temporal cornea (β = 0.51, *p* = 0.002) with no significant associations in the temporal limbus (β = 0.09, *p* = 0.30) or temporal conjunctiva (β = 0.07, *p* = 0.64). Similarly, there was a positive association between TMH and EIC density in the temporal conjunctiva (β = 0.42, *p* < 0.001), temporal cornea (β = 0.22, *p* = 0.004), and temporal limbus (β = 0.42, *p* < 0.001), but not in the central cornea, inferior whorl, or inferior cornea (all *p* ≥ 0.42). There were no significant associations between TBUT (β = −0.03, *p* = 0.76), NIBUT (β = −0.02, *p* = 0.85) and EIC density. Interaction terms between each of these parameters and location were also non-significant (interaction β range: −0.08 to 0.18; all *p* ≥ 0.25), indicating no location-dependent variation in these associations; therefore, results are presented as overall associations rather than location-specific effects. Similarly, there was no significant association between OSDI scores (β = −0.45, *p* = 0.49), MGS score (β = −0.07, *p* = 0.89), MGYLS score (β = −0.10, *p* = 0.47) and EIC density and none of the individual NRS symptom domains were significantly associated with EIC density at any location (all *p* ≥ 0.10).

The overall between-visit (day 1 morning–evening & day 1 morning–day 2 morning) correlation coefficients (Spearman’s rho) were ≥0.89 for all corneal locations, ≥0.57 for the temporal limbus, and ≥0.42 for the temporal conjunctiva.

## 4. Discussion

The study found no significant diurnal variation in EIC density or morphology at any locations. However, there was a distinct spatial distribution pattern, with higher EIC density and higher prevalence of medium to large EIC body size and dendritic characteristics, including EIC with long dendrites at the inferior cornea, temporal limbus, and temporal cornea, than other locations at all timepoints. These spatial variations in EIC distribution may have clinical relevance in DED, and they may be valuable to assess multiple corneal and conjunctival locations in addition to the central cornea, which has traditionally been the focus of EIC quantification in DED research. The absence of diurnal fluctuation suggests that the immune surveillance of the ocular surface in DED is relatively stable throughout the day. Clinically, this diurnal stability supports the utility of using EIC parameters as reliable biomarkers for assessing ocular surface inflammation, and timing is unlikely to influence measurements. By incorporating repeated imaging across several locations within 24 h, this study extends previous work that typically relied on a single timepoint and central corneal assessments.

The absence of significant diurnal variation in the present study is consistent with previous findings in healthy individuals [28,29]. Previous studies have similarly shown stable dendritic cell density and morphology across different timepoints of the day [28,29]. Although one study noted subtle changes in the proportion of morphologically mature cells upon awakening, such differences were not evident in the present DED cohort [28]. This discrepancy of findings between studies may reflect differences in immune cell classification criteria and morphological grading methods used between studies. The present study defined EIC based on a cell body size range of 10–65 µm and graded the highest morphological feature observed across five analyzed images per location. In comparison, the earlier study used a cell-level analysis with specific dendritic cell maturity criteria, which may account for the observed discrepancies.

DED symptoms, signs, and tear inflammatory markers have previously shown significant diurnal fluctuations [13,14,15,17,52]. Patients with DED report symptoms of dryness and discomfort, which are worse upon waking and in the evening [13,52]. Within-day variations in signs such as tear break-up time, tear osmolarity, ocular surface staining and tear meniscus height have been observed in both DED and healthy individuals [13,14,15,19,20,52,53,54]. However, in the current study cohort, there were no significant diurnal variations in DED symptoms and signs. Recruitment patterns of ocular surface immune cells, as studied through ocular wash samples, have shown diurnal variation in both healthy individuals and DED, during the sleep–wake cycle [55,56]. Neutrophils, granulocytes, and lymphocytes (T cells, Th17 cells) are recruited to the ocular surface in a circadian manner, with their concentrations peaking during nighttime eye closure [55,56,57,58]. In both DED and healthy individuals, a decline of inflammatory cells has been observed during open eye conditions [56]. Such intra-day variations have also been observed in some of the tear cytokines and chemokines in healthy individuals [10,12,16].

While studies on ocular wash and tear samples have shown diurnal variation in tear immune cell recruitment and tear cytokine levels, the current study found no diurnal variation in EIC using IVCM. This indicates a relatively stable intra-epithelial presence of immune cells throughout the day in DED. One possible explanation is that EICs located deeper in the epithelium (25–65 µm from the superficial epithelium) may not be directly influenced by eyelid closure, with changes during sleep potentially limited to the tear film or desquamated Cells. Diurnal symptom fluctuation in DED is often driven by changes in tear evaporation, or environmental exposure, which may not necessarily trigger rapid EIC changes detectable by IVCM. Alternatively, a decline in EIC numbers may be occurring, which rapidly recovers within the first hour after waking, as corneal hypoxia and sleep-related inflammatory stress resolve. This rapid clearance has been observed in healthy individuals and in DED, where the leukocyte count in the tear film significantly decreases shortly after awakening [56,57]. In the present study, all morning measurements were conducted within two hours of awakening; therefore, any immune response occurring immediately after sleep may not have been captured. Nevertheless, these considerations suggest that EIC features may represent a more stable biomarker of underlying immune status rather than short-term clinical fluctuation.

The topographical distribution pattern of EICs likely reflects location-specific immune surveillance activity at the ocular surface and may serve as a useful reference for interpreting EIC changes in DED. The distribution of EIC characteristics in DED aligns with previous observations of dendritic cell density and morphology in healthy individuals, albeit with higher EIC density and a greater prevalence of morphological features suggestive of inflammation [29,31,32,59,60,61].

Previous meta-analyses and cross-sectional studies in healthy individuals consistently showed higher immune cell density in the inferior and peripheral cornea compared with the central cornea [37]. Although the current DED cohort showed a similar topographical pattern, absolute density values were higher, highlighting the inflammatory nature of DED. The variability in EIC density has been reported in both healthy and DED populations and is likely driven by differences in immune cell definitions, image selection, quantification methods, and participant characteristics.

Importantly, prior evidence indicates that central corneal EIC density is generally higher in DED compared with healthy controls, with substantial heterogeneity across DED subtypes and disease severity [37]. Immune-mediated and more severe forms of DED tend to demonstrate greater EIC infiltration [37]. Taken together, the literature supports the presence of increased and spatially variable immune cell activity in DED, although the magnitude of change differs considerably between studies.

Evidence from previous DED cohorts also indicates that central corneal EIC density increases with disease severity and varies across DED subtypes [36,47,48]. EIC density was significantly higher than in healthy controls, with morphological changes observed early in mild DED (DEWS severity level 1), and showed higher central corneal EIC densities and morphological changes with increasing DED severity [47].

Subgroup comparisons further highlight significant heterogeneity in EIC density across DED subtypes. Aqueous-deficient DED and DED associated with underlying systemic immune conditions show markedly higher central corneal EIC density than evaporative or non-immune DED [48]. Aqueous-deficient DED had more than a threefold higher central corneal EIC density compared with evaporative DED [48]. A recent meta-analysis similarly showed that the mean difference in central corneal EIC density between healthy and DED groups was higher in individuals with mild–moderate DED symptoms, tear film break-up time of 6–10 s, and mild corneal staining [36]. Collectively, these findings suggest that EIC infiltration and morphological alterations are observable in early states of DED and more significant changes can be observed in more severe phenotypes.

The current study cohort had a central corneal mean EIC density of 45.6 cells/mm^2^, which aligns with values reported in several prior studies [36,62,63,64,65], although comparative data from other corneal regions remain limited. Direct comparison of results with historic datasets is limited by differences in study designs, imaging protocols, and definitions of dendritiform or epithelial immune cells [36]. Overall, existing evidence highlights that EIC changes in DED are heterogeneous, with different subtypes exhibiting distinct patterns and magnitudes of EIC alteration.

Large EIC cell bodies were more frequently observed in the current DED cohort than previously reported in healthy controls [29,49]. In healthy individuals, larger EICs are less commonly observed at the central cornea and inferior whorl, whereas in DED, they are more prevalent in these regions. Dendritic features suggestive of increased immune activity were also more frequent in DED, consistent with prior reports [29,49]. Similarly, large EICs with long dendrites were more commonly seen in the inferior cornea, temporal cornea, and temporal conjunctiva in DED participants. To some extent, this topographic distribution pattern has also been reported in ocular allergy and vernal keratoconjunctivitis, where allergy groups showed higher EIC density compared to healthy controls [27,30]. Taken together, these findings suggest that the spatial distribution pattern of EIC density and morphology is preserved in ocular surface inflammation. EICs in the limbal and peripheral cornea are believed to be more active and morphologically different to those in the central cornea [27,30,60,61,66,67], with a higher expression of MHC class-II and maturation markers on dendritic cells [67,68,69]. Experimental models show that peripheral corneal dendritic cells have a larger field area than central cells, and that inflammatory stimuli promote upregulation of MHC class II and co-stimulatory molecules, indicating local maturation and activation [67,68,69]. In both humans and animal models of DED, inflammation is marked by upregulation of HLA-DR and infiltration of Th1/Th17 cells into the conjunctiva and cornea [4,70,71]. Advances in IVCM further support the presence of both innate and adaptive immune cells within the cornea [46]. The greater prevalence of large, dendritiform EICs in the peripheral cornea likely reflects its proximity to the limbus and conjunctiva, which are rich in immune structures, vasculature, and lymphatics, facilitating immune surveillance and rapid cellular migration toward the central cornea during insult [4,46,67,68,70]. Higher EIC density in the inferior cornea in both DED and healthy eyes may relate to increased exposure to tear film mediators, mechanical stress from blinking, and environmental factors. Overall, while spatial and temporal patterns of EIC distribution appear preserved in DED, the increased density and altered morphology observed are consistent with chronic inflammation and heightened immune activity at the ocular surface.

EIC density was more repeatable at the central cornea (CoR ± 23.8 cells/mm^2^), temporal cornea (±27.5), and inferior whorl (±34.3) compared to the inferior cornea (±47.9), temporal conjunctiva (±42.3), and temporal limbus (±59.0). In comparison, in healthy eyes (using the same current study methods and authors, albeit in a younger cohort), CoR values of ±28.1 at the central cornea, ±25.6 at the inferior whorl, and ±10.2 at the bulbar conjunctiva have been reported, and higher variability was observed at the temporal cornea (±46.9) and temporal limbus (±56.4) [29]. Inter-observer repeatability of corneal dendritic cell density was reported previously using the same imaging protocol in healthy and allergic conjunctivitis showed higher variability in the allergic conjunctivitis group (CoR ± 12.0 at the corneal centre, CoR ± 14.3 at the temporal cornea, and CoR ± 19.1 at the conjunctiva, with higher values of CoR ± 29.1 and CoR ± 28.0 at the whorl and limbus, respectively) [30]. This indicates that biological variability in DED introduces greater measurement error and suggests that central, temporal cornea and inferior whorl may serve as reliable locations for monitoring EIC density changes over time. The better repeatability at these locations is likely because of a more consistent and uniform EIC distribution compared with the other regions and easier repositioning

The broader CoR range at peripheral locations in the current DED cohort indicates greater measurement variability at these sites. Repeatability is influenced by the underlying biological heterogeneity of EIC distribution, particularly at peripheral sites. This biological variability reflects true differences in EIC density across participants and locations. Such heterogeneity makes it difficult to relocate the exact same location during repeated imaging, particularly in the inferior cornea and in the limbus and conjunctiva, where there are no identifiable landmarks, thereby reducing repeatability.

No clear size effect was evident in the Bland–Altman plots at any location. Although systematic bias was minimal, the wide limits of agreement indicate substantial intra-subject variability in EIC density between visits, likely reflecting biological heterogeneity within the DED cohort. EIC density showed broad ranges across all regions, suggesting that inflammatory responses vary with disease severity and may not always be fully captured by density measures alone.

To improve consistency, baseline morning images were used to guide subsequent corneal imaging. However, precise relocation was not always achievable, mainly at the temporal cornea, limbus, and conjunctiva, due to fixation variability and difficulty identifying identical nerve patterns and landmarks. Given the known spatial variation in EIC distribution, positional differences within a region can substantially influence density estimates, contributing to broader CoR values and limiting sensitivity for detecting change.

Repositioning strategies, such as using the corneal whorl as a landmark and maintaining the same imaging plane, can enhance reproducibility. Visual alignment cues from the confocal image, fixation target, and tomocap position may further support consistent sampling. Despite these measures, biological and positional variability make it challenging to define diagnostic cut-offs based solely on CoR values. Rather, changes exceeding location-specific CoR thresholds are more likely to represent true biological change.

Importantly, strong correlations between visits for most corneal locations indicate stable inter-subject ranking despite intra-subject variability. This supports the utility of IVCM for group-level comparisons and longitudinal trend analysis, even where absolute repeatability is moderate.

Day-to-day agreement for EIC body size and other dendritic characteristics ranged from substantial to near perfect, remaining consistent within individuals across visits, similar to observations in healthy eyes [29]. This consistency enhances the reliability of IVCM for characterizing EIC behaviour in DED and supports its use in monitoring qualitative changes during disease progression or in response to treatment. Ocular surface staining and TMH were the two DED parameters significantly associated with EIC density in the current study. Higher staining grades and lower TMH values (lower tear volume) were associated with increased EIC density at some ocular surface locations. Ocular surface staining, a marker of inflammation, has shown significant correlations with EIC density in previous studies [23,47,72,73]. In previous studies, ocular surface staining emerged as the sole parameter positively associated with central corneal EIC density [36,47]. However, associations between EIC density and other DED parameters have been inconsistent across the literature, with some studies reporting significant correlations and others finding none [36]. TMH was positively associated with EIC density at the temporal conjunctiva, temporal cornea, and temporal limbus, but not at central or inferior corneal locations. Despite the proximity of the inferior cornea to the tear meniscus, EIC density at the inferior cornea and inferior whorl was not significantly related to TMH. Likewise, meibomian gland parameters (MGS and MGYLS) did not correlate with EIC density at any site, including the inferior cornea, where an association might be anticipated.

These findings suggest that local immune activation in DED does not consistently parallel structural or functional clinical parameters across all ocular surface regions. As the study was not powered to detect associations (*n* = 16), these results should be interpreted cautiously. Nonetheless, the consistent relationship between ocular surface staining and EIC density supports the role of staining as a marker of inflammation in DED.

This study has several limitations. First, while comparisons were made with data from healthy individuals, no healthy control group was included; instead, comparisons relied on historical controls from published literature. As a result, methodological and population characteristics differences across studies may affect comparisons or limit comparisons. Future work should include a matched non-DED healthy control cohort to enable definitive between-group comparisons and establishment of clinical thresholds. Secondly, the CoR findings for EIC density may not be generalizable to all DED populations, as participants with very low or very high EIC densities may exhibit different levels of repeatability. The sample size (*n* = 16) was powered to detect a within-subject mean change of 12 cells/mm^2^ with 80% power. This estimate was used for power calculation only and does not limit the magnitude of actual biological variability observed in the study. Indeed, Bland–Altman analyses showed that the observed mean differences between visits exceeded this value at several locations, reflecting heterogeneity as well as measurement variability. Therefore, the precision for agreement metrics such as CoR and LoA is moderate, and larger sample sizes would be expected to yield narrower LoA and more precise repeatability estimates. Despite these limitations, the study provides insights into the spatial and temporal characteristics of ocular surface immune cells in DED and highlights key considerations for future research and clinical interpretation.

Taken together, these results suggest location-specific EIC patterns and diurnal and day-to-day stability in DED, while establishing repeatability parameters to inform future studies. Given the smaller sample size and the absence of the inclusion of inflammatory biomarkers (e.g., tear cytokines, HLA-DR), this work should be viewed as confirmatory and feasibility-oriented, rather than definitive. Larger studies integrating inflammatory markers are warranted to define clinical thresholds and prognostic utility.

## 5. Conclusions

The findings confirm that DED exhibits diurnal stability in EIC density and morphology, and that the topographical distribution pattern previously observed in healthy individuals is preserved in those with DED. However, greater EIC density and morphology alterations were observed in DED compared to historical data from healthy controls. While larger absolute CoR values indicate intra-subject variability, the strong within-subject correlations support the reliability of IVCM for group-level comparisons and longitudinal trend analysis. These results highlight the potential of EICs as reliable biomarkers for DED diagnosis and assessing response to anti-inflammatory treatment. Larger studies are needed to establish precise clinical cut-offs and the prognostic value of EIC parameters.

## Figures and Tables

**Table 1:** Linear mixed model-estimated mean epithelial immune cell density (cells/mm^2^) and standard error (SE) by timepoint and location in 16 participants with dry eye disease.

Ocular Surface Location	Mean (SE) EIC Density (cells/mm^2^) in Day 1 Morning (8:00 a.m.–9:30 a.m.)	Mean (SE) EIC Density (cells/mm^2^) in Day 1 Evening (6:00 p.m.–8:00 p.m.)	Mean Difference (SE)	*p*-Value
Central cornea	26.8 (5.1)	26.5 (5.0)	0.3 (0.2)	0.84
Inferior whorl	38.3 (8.2)	38.5 (8.3)	−0.2 (0.2)	0.67
Inferior cornea	101.2 (21.7)	101.1 (21.6)	0.1 (0.2)	0.69
Temporal cornea	41.8 (9.1)	42.1 (9.2)	−0.3 (0.2)	0.72
Temporal limbus	104.3 (22.7)	104.5 (22.8)	−0.2 (0.2)	0.97
Temporal conjunctiva	43.1 (10.0)	43.2 (10.2)	−0.1 (0.2)	0.90

## Data Availability

Data are available upon reasonable request.

## Supplementary Materials

The following supporting information can be downloaded at: https://www.mdpi.com/article/10.3390/jcm15072582/s1, Table S1: Clinical characteristics of 16 dry eye disease participants at baseline (day 1 morning visit). Table S2: Epithelial immune cell density (cells/mm^2^) of 16 dry eye disease participants at baseline (day 1 morning visit). Table S3: Comparison of morphological features between day 1 morning and evening timepoints across six locations. The percentages of epithelial immune cell body size (um)—small, medium and large and dendritic characteristics are presented with the adjusted *p*-values obtained from Wald-z tests with Tukey’s adjustments. Table S4: Pairwise comparison of epithelial immune cell density (cells/mm^2^) between ocular surface locations. Model-Based aggregated means and standard error from the two timepoints (day 1 morning and evening) were used. The model-estimated mean difference and standard error of difference was calculated by back-transformation from the log scale. *p*-values were adjusted with Tukey’s adjustments. Table S5: Pairwise comparison of epithelial immune cell body size (small, medium and large) between ocular sur-face locations. Proportions of small, medium and large cells are aggregated from the two timepoints (day 1 morning and evening) for each location. *p*-values derived from Wald-z tests with Tukey’s adjustments. Table S6: Pairwise Comparisons of epithelial immune cell dendritic characteristics across ocular surface locations. The table presents *p*-values derived from Wald-z test with Tukey-adjusted post hoc comparisons for three different dendrite characteristics: presence of dendrites, presence of long dendrites, and presence of thick dendrites.

## Abbreviations

The following abbreviations are used in this manuscript:

DED	dry eye disease
IVCM	In vivo confocal microscopy
EIC	epithelial immune cell
OSDI	Ocular Surface Disease Index questionnaire
NIBUT	non-invasive tear break-up time
FBUT	fluorescein tear film break-up time
TMH	tear meniscus height
MGS	Meibomian Gland Secretion
MGYLS	Meibomian Glands Yielding Liquid Secretion
LLT	lipid layer thickness
CoR	coefficient of repeatability
