# Supplementary material for: Diurnal Variation, Topographical Distribution and Day-to-Day Repeatability of Ocular Surface Epithelial Immune Cells in Individuals with Dry Eye Disease
Source: J Clin Med. 2026 Mar 27;15(7):2582. doi: 10.3390/jcm15072582 (PMC13072989; doi:10.3390/jcm15072582)
Supplement: Supplementary file 1 [file jcm-15-02582-s001.zip › jcm-3937318-supplementary.pdf]

Journal of Clinical Medicine

MDPI

Supplementary data

Table S1: Clinical characteristics of 16 dry eye disease participants at baseline (day 1 morning visit).

Parameters	Mean (cells/mm²)	Standard error (SE)	Standard Deviation (SD)	Median (cells/mm²)	Interquartile range (25th-75th percentile)
**Age (years)**	49.4	4.2	16.2	45.5	38.0 - 66.2
**Duration of DED (months)**	54.5	8.2	30.9	48.0	36.0 - 61.0
**OSDI score (0-100)**	50.2	4.2	16.3	50.8	37.8 - 52.7
**NIBUT (seconds) (0-20)**	3.8	0.4	1.2	3.5	2.5 - 5.0
**FBUT (seconds) (0-10)**	2.9	0.4	1.0	3.0	2.0 - 4.0
**Central TMH (mm)**	0.17	0.00	0.03	0.17	0.14 - 0.20
**Oxford staining grade (0-5)**	1.3	0.02	0.6	1.5	0.4 - 1.9
**Bulbar conjunctival redness score (0-4)**	1.2	0.02	0.5	1.5	0.8 – 2.1
**MGS score (0-3)**	1.8	0.04	0.7	2.0	1.5 - 2.1
**MGYLS score (0-8)**	3.9	0.07	2.0	4.5	3.0 – 5.5
**LLT (um)**	64.4	5.6	25.5	66.0	44.7 - 84.2
**Presence of telangiectasia lower eyelid**	7/16 participants				
**Presence of lid wiper epitheliopathy lower or upper eyelid**	10/16 participants				
**Numerical rating scale for symptoms domains***					
**Dryness score (0-100)**	31.2	5.2	20.8	30.0	17.7 - 46.2
**Vision quality (0-100)**	75.4	7.9	31.5	90.0	60.5 - 100.0
**Fluctuating vision (0-100)**	77.0	7.7	30.9	93.5	67.7 - 100.0
**Itching (0-100)**	73.6	7.5	29.9	85.5	51.5 - 10.00
**Burning/Stinging sensations (0-100)**	34.5	6.8	27.4	42.5	20.2 - 67.2
**Foreign body sensations (0-100)**	58.6	7.2	29.0	67.5	42.0 - 82.0
**Eye pain (0-100)**	85.7	6.6	26.4	100.0	83.0 - 100.0
**Overall discomfort (0-100)**	34.4	6.0	23.9	34.0	18.0 - 51.2
**Systemic conditions**	Sjogren disease (2), coronary artery disease (2), fibromyalgia (2), dermatitis (1), hypertension (2), gastritis (2), systemic lupus erythematosus (1), hyperlipidaemia				

	(1), psoriasis (1), rheumatoid arthritis (1), IgA nephropathy (1), urticaria (1), polycystic ovary syndrome (1)

Footnote: OSDI: Ocular Surface Disease Index questionnaire; NIBUT: non-invasive tear break-up time; FBUT: fluorescein tear film break-up time; TMH: tear meniscus height; MGS: Meibomian Gland Secretion; MGYLS: Meibomian Glands Yielding Liquid Secretion; LLT: lipid layer thickness.

** For numerical rating scale for symptoms domains, a lower score means severe symptoms.*

Table S2: Epithelial immune cell density (cells/mm²) of 16 dry eye disease participants at baseline (day 1 morning visit).

**Locations**	**Mean (cells/mm²)**	**Standard error (SE)**	**Standard Deviation (SD)**	**Median (cells/mm²)**	**Interquartile range (25th-75th percentile)**
**Central cornea**	45.6	10.2	41.0	27.5	15.6 – 53.3
**Inferior whorl**	57.0	10.9	43.7	44.5	30.5 – 76.6
**Inferior cornea**	101.1	12.5	50.0	100.5	51.8 – 131.9
**Temporal cornea**	45.1	7.7	30.7	40.0	20.8 – 60.7
**Temporal limbus**	93.0	8.1	32.7	101.0	67.5 – 123.7
**Temporal conjunctiva**	40.5	5.3	21.1	38.8	11.0 – 64.4

Table S3: Comparison of morphological features between day 1 morning and evening timepoints across six locations. The percentages of epithelial immune cell body size (um) – small, medium and large and dendritic characteristics are presented with the adjusted p-values obtained from Wald-z tests with Tukey's adjustments.

**Locations**	**Morphological Feature**	**Day 1 morning (%)**	**Day 1 evening (%)**	**P value**
**Central Cornea**	Small body size	25.0	25.0	0.91
	Medium body size	43.8	37.5	
	Large body size	31.2	37.5	
	Presence of dendrites	75.0	70.0	1.00
	Presence of long dendrites	43.8	50.0	0.90
	Presence of thick dendrites	0.0	0.0	-
**Inferior Cornea**	Small body size	0.0	0.0	0.46
	Medium body size	5.0	0.0	
	Large body size	87.5	100.0	
	Presence of dendrites	100.0	100.0	1.00
	Presence of long dendrites	100.0	100.0	1.00
	Presence of thick dendrites	6.2	6.2	1.00
**Inferior Whorl**	Small body size	43.8	56.2	0.63
	Medium body size	26	31	

	Large body size	25.0	12.5	
	Presence of dendrites	56.2	50.0	0.91
	Presence of long dendrites	37.5	25.0	0.92
	Presence of thick dendrites	0.0	6.2	0.94
**Temporal Conjunctiva**	Small body size	0.0	0.0	-
	Medium body size	0.0	0.0	
	Large body size	100.0	100.0	
	Presence of dendrites	100.0	100.0	1.00
	Presence of long dendrites	91.7	91.7	1.00
	Presence of thick dendrites	58.3	58.3	1.00
**Temporal Cornea**	Small body size	0.0	0.0	0.59
	Medium body size	20.0	6.7	
	Large body size	80.0	93.3	
	Presence of dendrites	100.0	93.3	0.94
	Presence of long dendrites	73.3	80.0	0.97
	Presence of thick dendrites	6.7	13.3	0.91
**Temporal Limbus**	Small body size	0.0	0.0	-
	Medium body size	20.0	26.7	
	Large body size	80.0	73.3	
	Presence of dendrites	100.0	100.0	1.00
	Presence of long dendrites	86.7	80.0	0.90
	Presence of thick dendrites	100.0	100.0	1.00

Footnote: P-values were not computed for variables with no or minimal variation across timepoints, as statistical comparison was not possible.

Table S4: Pairwise comparison of epithelial immune cell density (cells/mm²) between ocular surface locations. Model-Based aggregated means and standard error from the two timepoints (day 1 morning and evening) were used. The model-estimated mean difference and standard error of difference was calculated by back-transformation from the log scale. P-values were adjusted with Tukey's adjustments.

**Location Comparison**	**Model-estimated mean difference (cells/mm²)**	**SE of difference**	**p-value**
**Central cornea vs. Inferior cornea**	- 74.7	22.3	**<0.001**
**Central cornea vs. Inferior whorl**	- 11.8	9.8	0.10
**Central cornea vs. Temporal conjunctiva**	- 16.6	11.3	0.07
**Central cornea vs. Temporal cornea**	- 15.3	10.5	0.11

**Central cornea vs. Temporal limbus**	-77.8	23.3	**<0.001**
**Inferior cornea vs. Inferior whorl**	62.9	23.2	**<0.001**
**Inferior cornea vs. Temporal conjunctiva**	58.1	24.0	**0.001**
**Inferior cornea vs. Temporal cornea**	59.4	23.7	**<0.001**
**Inferior cornea vs. Temporal limbus**	- 3.1	31.4	0.98
**Inferior whorl vs. Temporal conjunctiva**	- 4.8	13.0	0.99
**Inferior whorl vs. Temporal cornea**	- 3.5	12.1	1.00
**Inferior whorl vs. Temporal limbus**	- 66.0	24.3	**<0.001**
**Temporal conjunctiva vs. Temporal cornea**	- 1.3	13.6	0.99
**Temporal conjunctiva vs. Temporal limbus**	- 61.2	24.9	**<0.001**
**Temporal cornea vs. Temporal limbus**	- 62.5	24.8	**<0.001**

Table S5: Pairwise comparison of epithelial immune cell body size (small, medium and large) between ocular surface locations. Proportions of small, medium and large cells are aggregated from the two timepoints (day 1 morning and evening) for each location. p-values derived from Wald-z tests with Tukey's adjustments.

**Location Comparison**	**Large (%)**	**Medium (%)**	**Small (%)**	**p-value**
**Central cornea vs. Inferior whorl**	34.4 vs. 18.8	40.6 vs. 31.2	25.0 vs. 50.0	0.14
**Central cornea vs. Inferior cornea**	34.4 vs. 93.8	40.6 vs. 6.25	25.0 vs. 0.0	**<0.001**
**Central cornea vs. Temporal cornea**	34.4 vs. 86.7	40.6 vs. 13.3	25.0 vs. 0.0	**<0.001**
**Central cornea vs. Temporal limbus**	34.4 vs. 76.7	40.6 vs. 23.3	25.0 vs. 0.0	**<0.001**
**Central cornea vs. Temporal conjunctiva**	34.4 vs. 100.0	40.6 vs. 0.0	25.0 vs. 0.0	**<0.001**
**Inferior whorl vs. Inferior cornea**	18.8 vs. 93.8	31.2 vs. 6.25	50.0 vs. 0.0	**<0.001**
**Inferior whorl vs. Temporal cornea**	18.8 vs. 86.7	31.2 vs. 13.3	50.0 vs. 0.0	**<0.001**
**Inferior whorl vs. Temporal limbus**	18.8 vs. 76.7	31.2 vs. 23.3	50.0 vs. 0.0	**<0.001**
**Inferior whorl vs. Temporal conjunctiva**	18.8 vs. 100.0	31.2 vs. 0.0	50.0 vs. 0.0	**<0.001**
**Inferior cornea vs. Temporal cornea**	93.8 vs. 86.7	6.25 vs. 13.3	0.0 vs. 0.0	0.48

**Inferior cornea vs. Temporal limbus**	93.8 vs. 76.7	6.25 vs. 23.3	0.0 vs. 0.0	0.11
**Inferior cornea vs. Temporal conjunctiva**	93.8 vs. 100.0	6.25 vs. 0.0	0.0 vs. 0.0	0.50
**Temporal cornea vs. Temporal limbus**	86.7 vs. 76.7	13.3 vs. 23.3	0.0 vs. 0.0	0.50
**Temporal cornea vs. Temporal conjunctiva**	86.7 vs. 100.0	13.3 vs. 0.0	0.0 vs. 0.0	0.66
**Temporal limbus vs. Temporal conjunctiva**	76.7 vs. 100.0	23.3 vs. 0.0	0.0 vs. 0.0	0.71

Table S6: Pairwise Comparisons of epithelial immune cell dendritic characteristics across ocular surface locations. The table presents p-values derived from Wald-z test with Tukey-adjusted *post hoc* comparisons for three different dendrite characteristics: presence of dendrites, presence of long dendrites, and presence of thick dendrites.

**Comparison**	**Presence of dendrites p- value**	**Presence of long dendrites p- value**
**Central cornea vs. Inferior cornea**	0.22	**0.03**
**Central cornea vs. Inferior whorl**	0.57	0.68
**Central cornea vs. Temporal conjunctiva**	1.00	**0.01**
**Central cornea vs. Temporal cornea**	0.19	0.10
**Central cornea vs. Temporal limbus**	1.00	**0.04**
**Inferior cornea vs. Inferior whorl**	**0.01**	**0.01**
**Inferior cornea vs. Temporal conjunctiva**	1.00	1.00
**Inferior cornea vs. Temporal cornea**	0.99	1.00
**Inferior cornea vs. Temporal limbus**	1.00	1.00
**Inferior whorl vs. Temporal conjunctiva**	**0.03**	**0.001**
**Inferior whorl vs. Temporal cornea**	**0.02**	**0.003**
**Inferior whorl vs. Temporal limbus**	**0.03**	**0.001**
**Temporal conjunctiva vs. Temporal cornea**	1.00	0.68
**Temporal conjunctiva vs. Temporal limbus**	1.00	0.85
**Temporal cornea vs. Temporal limbus**	1.00	0.99
